# Structural and Thermal Examinations of Polyamide Modified with Fly Ash from Biomass Combustion

**DOI:** 10.3390/ma16155277

**Published:** 2023-07-27

**Authors:** Renata Caban, Adam Gnatowski

**Affiliations:** 1 Department of Materials Engineering, Faculty of Production Engineering and Materials Technology, Czestochowa University of Technology, 42-201 Czestochowa, Poland; 2 Department of Technology and Automation, Faculty of Mechanical Engineering and Computer Science, Czestochowa University of Technology, 42-201 Czestochowa, Poland; adam.gnatowski@pcz.pl

**Keywords:** polymer composites, thermal properties, polyamide 6, fly ash, crystallinity

## Abstract

This paper presents the results of examinations of the structure and crystallinity of polyamide (PA6) modified with fly ash from biomass combustion in a fluidized-bed boiler. Composites based on a PA6 matrix were examined. They contained 5, 10, and 15 wt% fly ash. Fourier-transform infrared with attenuated total reflectance spectroscopy (FTIR-ATR) was used to identify the characteristic functional groups present in the chemical structure of polyamide and composites based on its matrix. Structural analysis was performed using a differential scanning calorimeter (DSC) and microscopic examinations. Analysis of the values of thermal effects determined using the DSC technique allowed for the evaluation of the degree of crystallinity of the materials studied. Polyamide is usually considered to be a two-phase system consisting of crystalline and amorphous regions. The addition of the filler in the form of fly ash reduced the degree of crystallinity of the studied specimens. Based on the FTIR-ATR spectra and the recorded DSC curves, it was found that the α-phase was the dominant crystalline phase in the studied materials. Microscopic examinations were conducted to analyze the microstructure of the materials, providing information on the distribution and shape of the filler particles. Most of the particles ranged in size from a few to tens of micrometers. Furthermore, the use of scanning electron microscopy coupled with energy-dispersive X-ray spectroscopy (SEM–EDS) allowed for the analysis of the distribution of chemical elements in selected filler particles.

## 1. Introduction

In polymeric materials, the structure can be easily modified physically, which at the same time determines how they can be used. One method for the aforementioned alteration is the introduction of disparate types of fillers: organic, inorganic, and others, including waste [1,2,3]. Since the 1980s, the disposal of waste generated in the energy industry has been identified as a very important issue. Energy waste, both generated at energy facilities and deposited in landfills, is a valuable source of raw materials and mineral products [4]. Increasingly, biomass from agricultural crops, wood industry waste, or industrial waste is being used as an energy fuel. It has also been observed that thermal energy is obtained by co-firing fossil fuels, mainly coal and biomass. The advantage of biomass combustion is its zero emissions of carbon dioxide, since, during combustion, as much carbon dioxide is emitted as the biomass will take up during the vegetation. However, some problems remain unsolved, including emissions of gases associated with oxygen deficiency in the combustion process, such as carbon monoxide, polycyclic aromatic hydrocarbons (e.g., xylan), aldehydes, or other chlorine-related organic compounds (e.g., methyl chloride), and the formation of fly ash during combustion [5]. Fly ash from the combustion of conventional fuels, such as brown and hard coal, is a commonly used additive for cement composites [6]. Fly ash from biomass combustion generally does not meet the standard requirements for ash as an additive in the production of building materials and in road construction. It is therefore necessary to look for other directions of its use [7,8]. Fly ash is more economical and ecological than traditional mineral fillers, so it is an attractive alternative as a polymer reinforcement. Recently, there has been increasing interest in fly ash among plastic processors, including using them as fillers for polymers such as polypropylene (PP), polyvinyl chloride (PVC), polyethylene (PE), and polyethylene terephthalate (PET). The use of fillers in polymer material processing technologies is environmentally friendly, as it helps reduce the volume of waste, and economical, contributing to lower product manufacturing costs. Fillers in the form of fly ash generally enhance mechanical properties, increase abrasion resistance, diminish the flammability of polymers, or change the density of plastics [9,10]. The process of filling polymeric materials with fly ash is carried out in such a way that they largely retain the characteristic properties of the initial material or improve them and can perform specific functions to a certain extent. The only limitation of using fly ash as a filler is its color, as it does not allow the products to reach a degree of whiteness [11]. The value of fly ash as a reinforcement in polymer composites has already been the main topic of many studies, and several authors have analyzed the use of fly ash as a reinforcing material for polymers such as polypropylene [12], polyamide [13,14], polyurethane [15], or low-density polyethylene [16]. This paper displays the results of examinations of polyamide modified with fly ash sourced from the combustion of biomass in a fluidized-bed furnace. Polyamide 6 (PA6) is a partially crystalline thermoplastic material. Polyamide macromolecules are built from a repeating amide group (-CO-NH-) that is present in the molecular structure of the polymer. There are hydrogen bonds between the amide groups, determining a number of their properties. In polyamides, the carbonyl oxygen of the amide performs as a hydrogen-bond acceptor, and the -NH group acts as a hydrogen-bond donor [17]. Polyamide is usually considered to be a two-phase system consisting of crystalline and amorphous regions. The crystalline phase of polyamide 6 can be present in three polymorphic varieties: the monoclinic α variety, the metastable β form, and the hexagonal γ variety. These phases usually coexist in different amounts, which are related to the requirement for a maximum number of H bonds [18]. In the α and γ forms, the molecular chains are organized in parallel sheets, stabilizing the hydrogen bonds. The polyamide structure is dominated by the stable monoclinic α-phase, which has an extended planar chain conformation. The H bonds lie between the parallel but oppositely directed chains. In the γ form, the hydrogen bonding occurs between parallel chains in adjacent sheets, rather than within the sheets. The γ form is unstable and can be transformed into the α form by the annealing process [19]. The β form is metastable and is considered to be an intermediate structure with a variable degree of disorder [20]. Polyamide 6 is one of the most important engineering polymers. It is characterized by good mechanical, functional, and technological properties. The application of filler in the form of fly ash induces changes in the functional properties of polyamide. The properties of a polymer composite are determined by the properties of its individual components, the internal structure of the polymer matrix, and the kind of reinforcing phase. Assessing the structure at the chain microstructure, elemental cell, and supramolecular levels requires research using several techniques to characterize the structure (both qualitatively and quantitatively) and assess its impact on the performance of the composites. An analysis of the available literature shows that Fourier-transform infrared spectroscopy makes it possible, among other things, to assess the structure at the molecular level, and also to identify the crystalline forms of the polymer [17,18,19,20,21,22,23,24]. Differential scanning calorimetry allows the determination of the degree of crystallinity, and also the characterization of thermal parameters such as melt temperature and crystallization temperature [9,11,25,26,27]. In this paper, we aimed to explain the influence of fillers, in the form of fly ashes, on the properties of polyamide 6. Fly ashes are waste products, cheap and easily accessible, and their amount increases together with the continuing activity of the power industry based on coal. The main objective of modifying polyamide with fly ash was to obtain a new, cheaper structural material, and to analyze the changes in thermal and structural properties affecting performance, allowing for wider application of the obtained material. Predicting properties and conditions plays an important role in planning compositions and obtaining polymer products. This paper presents the results of a study of the effect of the addition of fly ash from the combustion of biomass in a fluidized-bed furnace on the structure and crystallinity of specimens made of polyamide 6. The research included spectroscopic structural analysis (Fourier-transform infrared with attenuated total reflectance spectroscopy), thermal analysis (DSC), and microscopic examinations.

## 2. Materials and Experimental Procedure

**Figure 1:**
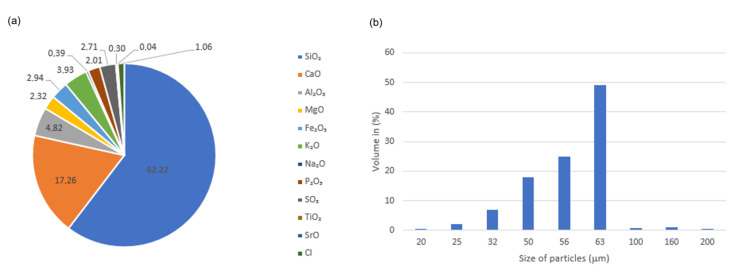
Chemical composition (**a**) and particle size distribution (**b**) of fly ash from biomass combustion.

This paper displays the results of examinations of polyamide 6 (PA6) composites modified with fly ash from biomass combustion in a fluidized-bed boiler. Polyamide 6 (PA6) with the commercial name Tarnamid T-27, synthesized by Zakłady Azotowe (Tarnów, Poland), was used as the matrix of the composites. Fly ash produced by GDF Suez Energia Polska S.A. (Polaniec, Poland), obtained from the combustion of biomass containing 80% wood waste and 20% coconut shells in a fluidized-bed furnace, was used as a filler. The chemical composition of the fly ash and the particle distribution are shown in Figure 1.

The polyamide was dried in a thermal chamber at 80 °C for 24 h before processing. To increase the compatibility of the polymer matrix and filler, a silane formulation was applied to the fly ash and polyamide. A 20% water solution of aminosilane type A 1100 was used, which is a binding agent and causes an increase in adhesion.

The silane-treated polyamide and fly ash were dried under normal air pressure before the extrusion process in a Shini CD-9-CE cabinet dryer (Shini Plastics Technologies, Inc., New Taipei, Taiwan) at 80 °C for 12 h. Composites with 5, 10, and 15% fly ash contents by weight were made by extrusion molding. A single-screw extruding press with a Rolbatch SJ45 straight extrusion head (Rolbatch, Eberswalde, Germany), equipped with a compound dosage system and a degassing system, was used to perform the composite extrusion molding. The extrusion parameters were as follows: cylinder heating temperature of zone I: 240 °C, zone II: 255 °C, zone III: 265 °C; temperature of the extrusion head: 270 °C; auger rotation: 160 min^−1^. The material was then milled using a Shini SG-2417-CE slow-speed mill (Shini Plastics Technologies, Inc., New Taipei, Taiwan). The test specimens were prepared using injection molding technology on a KraussMaffei KM65-160C1 (KraussMaffei Group GmbH, Munich, Germany) injection molding machine. The following processing parameters were used: injection pressure—90 MPa, clamping pressure—50 MPa, clamping time—20 s, time of cooling—20 s, mold temperature—100 °C, melt temperature—280 °C.

The percentages of fly ash content and symbols of the individual specimens are presented in Table 1.

The chemical structure of polyamide and polyamide composites with a fly ash filler was examined using a Shimadzu Irraffinity-1s Fourier-transform infrared spectrophotometer (FTIR) (Shimadzu Corporation, Kyoto, Japan) and the method of attenuated total reflectance (ATR). The spectra were registered in the measurement range of 400 to 4000 cm^−1^ at 60 scans per measurement. The resolution of the camera was 2 cm^−1^. Differential scanning calorimetry tests were conducted using a Netzsch PC 200 scanning microcalorimeter (Netzsch, Germany). The mass of the specimens ranged from 6 to 10 mg. DSC thermograms were documented while the specimens were heated at a rate of 20 °C/min over a temperature range of 30 to 275 °C. The specimens for DSC testing were cut perpendicular to the direction of plastic flow in the mold from the specimens obtained by injection molding. This minimized the skin–core effect. Analysis of the DSC thermograms was carried out using Netzsch PC 200 6.1 software. This software allowed for the examination of the specimens’ melting profiles in the given temperature range and the determination of the surface area between the thermographic curve and the basic line in the range of endothermic reflex. Based on the recorded melting enthalpy, with respect to the percentage of filler content, the values of the degree of crystallinity were adjusted from the following relationship:

(1) $$S_{K}=\frac{\Delta H_{p}}{w_{c}\Delta H_{k}}\cdot 100\%$$

where

*S*_K_—degree of crystallinity (%);
Δ*H_p_*—the enthalpy of fusion for the material examined (J/g);
Δ*H_k_*—the enthalpy of fusion for the entirely crystalline material (J/g);
*w*_c_—the mass fraction of homopolymer added to the composite studied.

The melting enthalpy of the crystallographic forms of PA6 had similar values (the Δ*H_k_* of the α form was 241 J/g, while the Δ*H_k_* of the γ form was 239 J/g), so the mean enthalpy value used for the calculations was 240 J/g [27]. A Keyence VHX-7000 digital optical microscope (Keyence Corporation, Osaka, Japan) and a JEOL JSM-6610 LV (JEOL Ltd., Tokyo, Japan) scanning electron microscope, equipped with energy-dispersive spectroscopy (EDS), were used to observe the microstructure of the specimens. These examinations were used to analyze the microstructure of the materials and the distribution and shape of the filler particles. Furthermore, the use of an SEM-EDS device allowed for the analysis of the distribution of chemical elements in selected filler particles.

## 3. Results and Discussion

**Figure 2:**
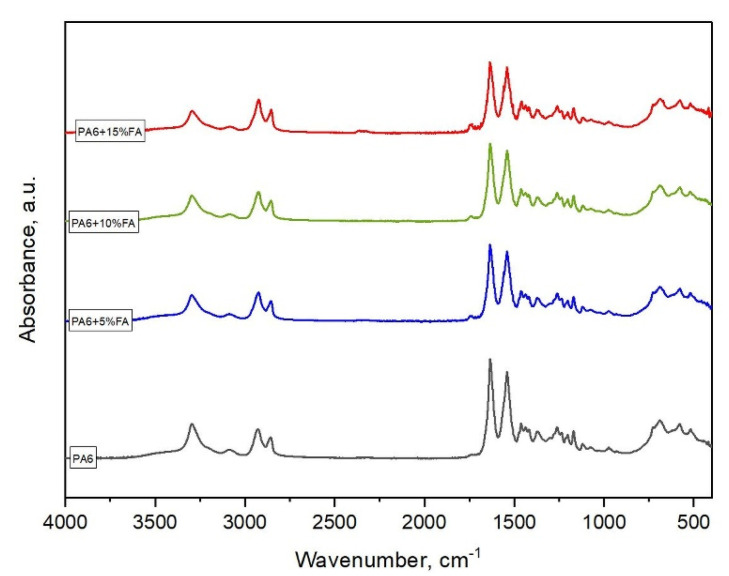
Fourier-transform infrared with attenuated total reflection (FTIR-ATR) spectra of the studied materials.

The spectroscopic (FTIR-ATR) results of polyamide and polyamide composites with different fly ash contents are shown in Figure 2.

Study of the FTIR-ATR spectra made it possible to identify characteristic absorbing groups present in the chemical structure of polyamide 6 and PA6 matrix composites with different amounts of filler in the form of fly ash obtained from biomass. Each absorbing group was assigned in the IR spectrum to an appropriate absorbing peak with specific wavenumber values.

All peaks of the FTIR spectrum of PA6, along with their assignments based on the available literature data [23,24], are shown in Table 2.

PA6 is a semi-crystalline polymer that can form crystals in stable α and γ forms and an unstable β form. PA6 contains (CH_2_)_5_ segments, distinguishable by a parallel or antiparallel arrangement of secondary amide groups (NH-CO). These amide bonds cause hydrogen bonding between the chains and form a planar surface [28]. In the FTIR-ATR spectra of PA6 and composites based on its matrix, the bands associated with the amide groups are primarily formed by the mutual arrangement of hydrogen bonds, while the vibrations originating from the methylene groups are related to the chain conformation. FTIR-ATR spectroscopy is a research technique that is also used to identify the crystalline forms present in the polymer matrix. The absorption bands recorded at 838, 929, 960, 1025 and 1200 cm^−1^ (Table 2, Figure 2) indicate that PA6 principally consists of the crystalline phase α. The presence of a small amount of the crystalline form γ is evidenced by the presence of a band at about 976 cm^−1^ [17]. The band at 3300 cm^−1^ comes from the stretching vibration of the N-H hydrogen bond (amide A) in the crystalline phase, while the band at 3085 cm^−1^ comes from the Fermi resonance of the N-H stretching vibration and the first overtone of the amide II band. The position of this band depends on the strength of the hydrogen bonds. The amide B band comes from the stretching vibrations of the N-H groups associated with the hydrogen bond. The spectral feature at 1633 cm^−1^ is associated with stretching vibrations of the C=O carbonyl group (amide I), while mixed movements of C-N stretching vibrations and bending vibrations in the N-H bond plane occur in the band at 1540 cm^−1^ (amide II). The two bands occurring at 2934 cm^−1^ and 2865 cm^−1^ correspond to asymmetric and symmetric C-H stretching vibrations, respectively [29,30]. The bands at 1473 cm^−1^ and 1421 cm^−1^ are due to CH_2_ scissor vibrations of -NH groups and -CO groups, respectively. The bands at 1462 cm^−1^ and 1370 cm^−1^ correspond to the scissor and torsional vibrations of CH_2_, respectively. The latter four bands are characteristic of the *trans* conformation of the polymer chain fragments. However, the presence of a band located at 1436 cm^−1^, corresponding to the bending vibration of the CH_2_ group next to the amide nitrogen atoms, indicates a rotational deviation of the -CH_2_-CONH-CH_2_- group from the ideal *trans* conformation. The band appearing at 1168 cm^−1^ corresponds to vibrations of the N-C-O group [31,32].

**Figure 3:**
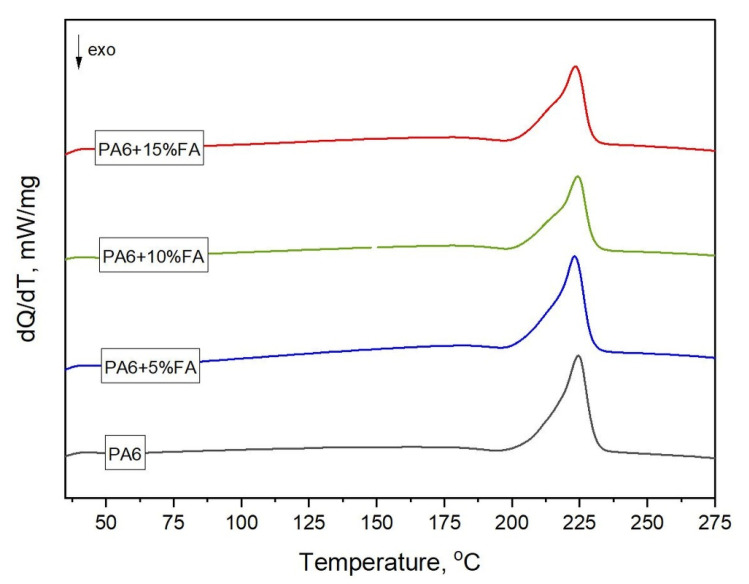
DSC thermograms of the specimens (melting curves).

There are many research papers in the literature on the analysis of the effects of various additives (fillers) introduced into polymers on their structure, as well as on the values of characteristic temperatures [33,34,35]. Differential scanning calorimetry (DSC) was used to determine the changes occurring in the thermal properties of the materials studied and their crystalline structure (degree of crystallinity), caused by the effect of the filler (fly ash). Figure 3 and Table 3 show the results of tests using differential scanning calorimetry.

The configuration of hydrogen bonds formed between the amide groups of neighboring macromolecules has a very significant effect on the crystal structure of PA6. When analyzing the crystallinity of this polymer, it is important to take into account crystallographic polymorphism, i.e., the potential to form different variations of the space lattices. PA6’s crystalline phases differ in the degree and quality of their arrangement. On the recorded DSC curves of all of the tested specimens, a melting peak was observed at about 224 °C, corresponding to the melting of the α-phase crystals. This phase was the dominant crystalline phase in the materials studied. Two interesting features were observed for PA6 specimens modified with 5, 10, and 15 wt% fly ash: Firstly, in addition to the higher peak recorded at about 224 °C, the addition of fly ash produced a lower endothermic peak. This resulted in the presence of a slight inflection in the melting curve at about 210 °C, which was associated with the melting of less-stable crystals of the γ form. This effect was less pronounced in the DSC curve recorded for unmodified PA6. Secondly, it can be seen that the addition of 15 wt% fly ash caused the melting point of the α-phase to shift slightly towards lower temperatures. This may indicate that the addition of fly ash promotes the transformation of α → γ, while possibly destroying the perfection of the α-phase crystals [36]. The presence of a small amount of the γ-phase in the specimens was confirmed by FTIR-ATR spectroscopy examinations (Table 2). A similar phenomenon was observed by Brzozowska-Stanuch et al. [37], who studied the effect of the addition of multiwalled carbon nanotubes on the PA6 matrix, and by Legocka et al. [38], who analyzed the effect of modified halloysite on the structure and thermal and mechanical properties of polyamide 6. The results obtained indicate an increased ability of composites with lower filler content to crystallize. The increase in the crystalline phase documented for the specimens made of polyamide 6 with a 5% filler content was likely induced by the formation of heterogenic nucleation centers around the filler during cooling in the injection mold. Polyamides filled with more fly ash particles (PA + 10% FA, PA + 15% FA), on the other hand, had a lower degree of crystallinity than unmodified PA6. A higher filler content reduces nucleation and the possibility of further growth of the crystalline phase of the polymer matrix. It is reasonable to assume that the filler particles impede the diffusion of macromolecule segments into the growing crystallites, resulting in the formation of a smaller volume of the crystalline phase. The introduction of some fillers into the polymer matrix increased the crystallization temperature in the matrix, reducing the size of the crystallites and the amount of the crystalline phase. At the same time, these fillers acted as a heterogeneous nucleating agent [39].

**Figure 4:**
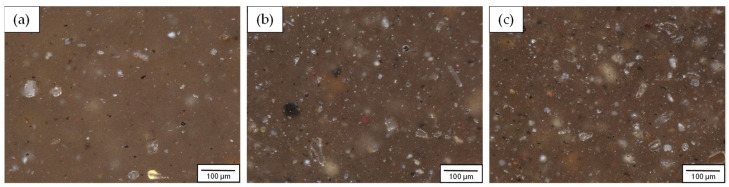
Microstructures of polyamide 6 composites with varying fly ash contents; magnification 400×: (**a**) PA6 + 5% FA, (**b**) PA6 + 10% FA, (**c**) PA6 + 15% FA.

Structural examinations of PA6 composites with different fly ash contents were performed using a digital optical microscope. Observations were carried out on metallographic sections at 400× magnification. The microstructures obtained for the materials studied are presented in Figure 4.

**Figure 5:**
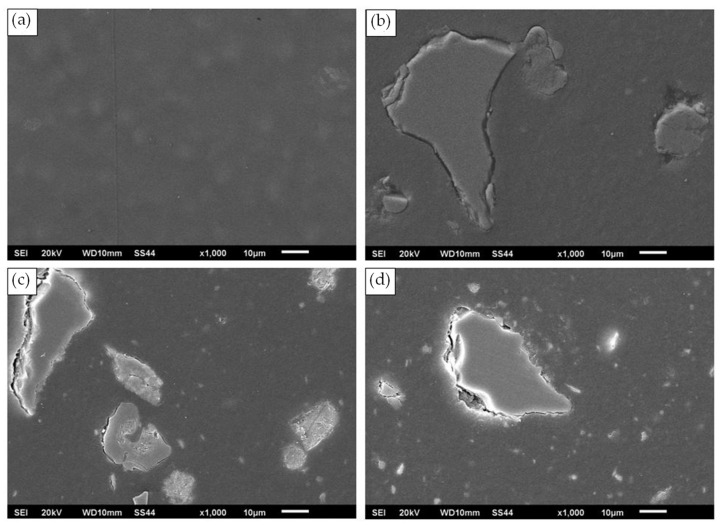
SEM microstructures of the materials studied: (**a**) PA6, (**b**) PA6 + 5% FA, (**c**) PA6 + 10% FA, (**d**) PA6 + 15% FA.

An even distribution of fly ash particles in the polymer matrix and the absence of conglomerates were observed. The size, shape, and color of the filler particles varied widely. The largest particles did not exceed 100 µm. However, the vast majority of the particles had dimensions of a few to tens of micrometers. The color of the filler particles observed in images of the microstructures of polyamide matrix composites may depend on the oxides present. The filler particles are characterized by a light to dark gray and light brown color and consist predominantly of silicon, aluminum, and iron oxides [40]. For an in-depth analysis of the structure, studies were also performed on a JEOL JSM-6610 LV scanning microscope. The microstructures obtained for the materials studied are presented in Figure 5.

The fly ash grains observed in the microstructures of the tested composites had irregular and elongated shapes, and their size did not exceed 100 µm (Figure 5b–d). Particle morphology largely depends on the type of fuel used and the method of combustion [39]. The ash grains formed in conventional boilers are spherical in shape, while fly ash from fluidized-bed boilers forms particles with very irregular shapes and elongated edges [16,41,42].

**Figure 6:**
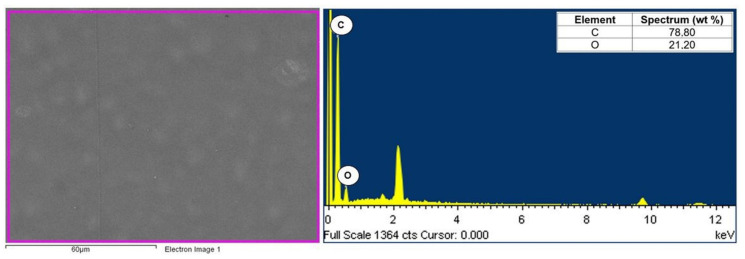
Area and spectrum of SEM-EDX analysis for PA6.

**Figure 7:**
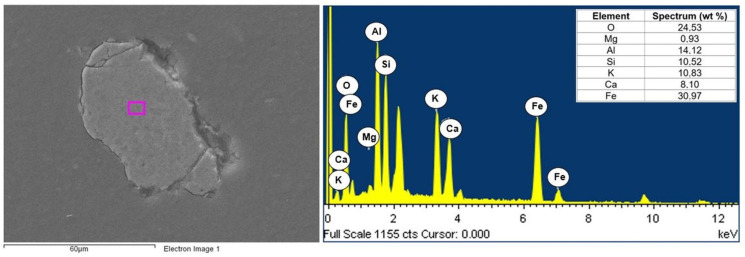
Area and spectrum of EDX analysis for PA6 + 5% FA.

**Figure 8:**
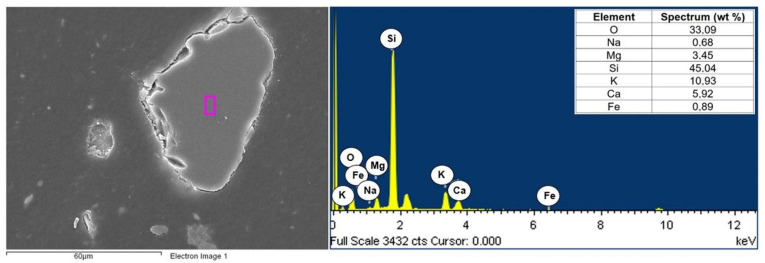
Area and spectrum of SEM-EDX analysis for PA6 + 10% FA.

**Figure 9:**
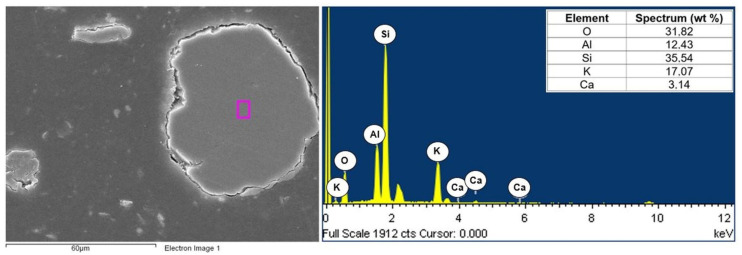
Area and spectrum of SEM-EDX analysis for PA6 + 15% FA.

The composites consisted of two phases of polyamide (PA6) and fly ash. A JEOL JSM6610LV scanning microscope with an EDS attachment was used to visualize the morphology of the composite components. The analysis concerned the chemical composition of the matrix (polyamide specimen) and selected filler particles in polyamide matrix composites with different contents of fly ash (5, 10, and 15%). Examples of the chemical composition of polyamide (PA6) are shown in Figure 6, while the chemical composition of selected fly ash particles is shown in Figure 7, Figure 8 and Figure 9.

Calcium, magnesium, aluminum, iron, and silicon compounds were present in the form of oxides (SiO_2_, Al_2_O_3_, Fe_2_O_3_, CaO, and MgO), as evidenced by the significant proportion of oxygen in the elemental composition of fly ash produced from biomass combustion (Figure 7, Figure 8 and Figure 9). Fly ash from the combustion of plant biomass contains more oxides (e.g., CaO, K_2_O, and P_2_O_5_) compared to other fuels [43]. Information on the chemical composition of ashes from the combustion of a particular type of plant is an important element in assessing their potential recovery or the possibility of evaluating their suitability as starting materials for the creation of composite materials. The chemical composition of fly ash determines its functional properties and, thus, the directions of its application. The use of fly ash as a filler in polymer composites can contribute to reducing the adverse environmental impact of fly ash itself. Biomass is considered to be a zero-emission fuel and is increasingly used as the base fuel in power plants. Byproducts of the combustion of biomass are characterized by a different chemical and phase composition compared to the combustion of brown or hard coal [5].

## 4. Conclusions

Analysis of the FTIR-ATR spectra made it possible to identify characteristic absorbing groups present in the chemical structures of polyamide 6 and PA6 matrix composites with different amounts of filler in the form of fly ash obtained from biomass. The absorption bands occurring at wavenumbers 838, 929, 960, 1025, and 1200 cm^−1^ showed that PA6 consists predominantly of a crystalline α-phase. The presence of a small amount of the γ crystal form of PA6 was evidenced by the presence of a band at about 976 cm^−1^. On the recorded DSC curves of all of the tested specimens, a melting peak was observed at about 224 °C, corresponding to the melting of the α-phase crystals. This phase was the dominant crystalline phase in the materials studied. Based on the results obtained by the DSC method, it was found that the introduction of fly ash into the polymer matrix caused a decrease in the degree of crystallinity. Polyamides filled with more fly ash particles (PA6 + 10% FA, PA6 + 15% FA), on the other hand, had a lower degree of crystallinity than unmodified PA6. It is reasonable to assume that the filler particles impede the diffusion of macromolecule segments into the growing crystallites, resulting in the formation of a smaller volume of the crystalline phase. An even distribution of fly ash particles in the polymer matrix and the absence of conglomerates were observed. The size, shape, and color of the filler particles varied widely. The largest particles did not exceed 100 µm. However, the vast majority of the particles had dimensions of a few to tens of micrometers. The fly ash grains observed in the microstructures of the tested composites had irregular and elongated shapes. Calcium, magnesium, aluminum, iron, and silicon compounds were present in the form of oxides (SiO_2_, Al_2_O_3_, Fe_2_O_3_, CaO, and MgO), as evidenced by the significant proportion of oxygen in the elemental composition of fly ash produced from biomass combustion. The use of fly ash from biomass combustion as a filler makes it possible to obtain a cost-effective filler compared to those used in industry today. At the same time, it contributes to reducing the amount of waste generated during biomass combustion. The results of this study offer the possibility of using polyamide composites in various industries as raw materials for a variety of engineering components.

## Figures and Tables

**Table 1:** Designations of specimens used in the study.

Specimen	PA6	PA6 + 5% FA	PA6 + 10% FA	PA6 + 15% FA
Content of fly ash, %	-	5	10	15

**Table 2:** Band location and FTIR spectrum assignment of specimens used in the study.

Band Location, cm^−1^	Assignment	Crystalline Phase
690	C-C bending	
730	CH_2_ waging	
838	CO-NH in plane	α-Crystalline
929	CO-NH in plane	α-Crystalline
960	CO-NH in plane	α-Crystalline
976	CO-NH in plane	γ-Crystalline
1025	CO-NH in plane	α-Crystalline
1061	C-C stretching	
1168	N-C-O groups/CH_2_ twisting	
1200	C-CH bending (sym.) CH_2_ twisting	α-Crystalline
1261	C-N stretching (amide III)	
1370	CH_2_ twisting	
1421	CH_2_ scissoring next to C=O group	
1436	CH_2_ bending vibration next to N-H group	γ-Crystalline
1462	CH_2_ scissoring	
1473	CH_2_ scissoring next to N-H group	
1540	C-N stretching and N-H bending of hydrogen-bonded N-H groups (amide II)	
1633	C=O stretching (amide I)	
1734	N-H/CO overtone	
2856	CH_2_ stretching (sym.)	
2924	CH_2_ stretching (asym.)	
3085	Fermi resonance of N-H stretching with the overtone of amide II modes (amide B)	
3300	Hydrogen-bonded N-H stretching in crystalline phase (amide A)	

**Table 3:** The results of DSC investigations collected from calculations of the Netzsch Proteus program.

Polymer	Degree of Crystallinity (%)	Melting Range (°C)	Max. Melt Temperature (°C)
PA6	20.74	217.3–227.4	224.0
PA6 + 5% FA	21.80	216.0–227.3	224.0
PA6 + 10% FA	19.86	218.6–226.4	224.2
PA6 + 15% FA	19.65	218.3–225.7	223.4

## Data Availability

Data are contained within the article.
